# Supplementary material for: Diagnostic performance of the GenoType MTBDRplus VER 2.0 line probe assay for the detection of isoniazid resistant Mycobacterium tuberculosis in Ethiopia
Source: PLoS One. 2023 Apr 26;18(4):e0284737. doi: 10.1371/journal.pone.0284737 (PMC10132600; doi:10.1371/journal.pone.0284737)
Supplement: Attachment — Submitted filename: Comment to Diagnostic performance of the GenoType MTBDRplus VER 2.0 LPA PLOS ONE.docx [file pone.0284737.s002.docx]

To the editors:

**Summary**

 This manuscript assesses the Diagnostic performance of the GenoType MTBDRplus VER 2.0-line probe assay for the detection of isoniazid resistant Mycobacterium tuberculosis in Ethiopia.

 The subject remains a point of interest, however looking into the current form the manuscript seems to have major needed correction, mostly clarifying and updating the mix-ups between the methods and result section. The discussion part is well written, and a few minor edits are required there too.

**Major comments**

 Line 3 and 10. Order of the co-authors, we cannot have ‘e’ before ‘b’. Please replace ‘e’ with ‘b’ and correct the order accordingly.

**Abstract:**

Line 27. Introduce version 1.0 here and clarify why for version 2 LPA. Suggested reference: [R. N. Yadav](https://pubmed.ncbi.nlm.nih.gov/?term=Yadav%20RN%5BAuthor%5D) et al [Tuberc Respir Dis (Seoul).](https://www.ncbi.nlm.nih.gov/pmc/articles/PMC8273024/) 2021 Jul. You may also include this part in the ‘introduction’ rather than in the abstract.

 Line 29. 137 is a result, should not come here in the methods. In the methods readers are interested ‘the how part of the procedure and selections of participants.

 Line 35. To include your 137 here, the suggestion might look like – ‘A total of 137 isolates were identified, of those 62 were Hr-TB, 35 multidrug resistant tuberculosis (MDR-TB) and 40 INH susceptible. Remove ‘were evaluated’ this is more to go in the methods part.

 Line 43. Replace ‘improved’ with higher since there was no intervention to claim this. Even ‘higher’ may only be used if supported by a significance test. Inference like high or low may not be used without statistical significance backing it up. P value or confidence interval, as appropriate.

 Line 48. In your keywords. You may remove ‘isoniazid resistant M tuberculosis’. It does not add value while it is redundant. Adding ‘Ethiopia’ does not seem to add that much too. Unless you insist better without it.

**Introduction**

See the comment in the abstract on this: Introduce version 1.0 and clarify why for version 2 LPA. Suggested reference: [R. N. Yadav](https://pubmed.ncbi.nlm.nih.gov/?term=Yadav%20RN%5BAuthor%5D) et al [Tuberc Respir Dis (Seoul).](https://www.ncbi.nlm.nih.gov/pmc/articles/PMC8273024/) 2021 Jul. You may also include this part in the ‘introduction’ rather than in the abstract.

 Line 81-84. Please align this description of aim with that of the abstract and make it clearer as it seems not immediately clear on its consistency, especially with the abstract.

**Methods**

Line 86. On the 137, see the same comment under abstract.

Line 90-103. This part is not really a method to address your objective, probably for the survey. Thus, it should be removed. Here, you rather describe in a succinct way the exact methods/procedures you used to help you reach to 137 (without mentioning numbers, unless required). For instance, the first paragraph of your result is mostly the methods part that should come in this section.

 Line 136-140. Sensitivity against what? Phenotypic DST? Why do you want to measure it against weak methods, at least it is not a gold standard. If there was no alternative way to measure the test (Genotype) performance, then it should be stated as a limitation in the discussion part. I haven’t seen this in the discussion.

**Results**

Line 150. Include 1594 with the denominator in your abstract too, e.g., 62% (1594/2268).

*Suggestion: ‘Phenotypic DST results of MTBC isolates were available for 62% (1594/2268) of those 3.9% (62/1594) and 2.2% (35/1594) were from Hr-TB and MDR-TB, respectively.’*

 Line 153-156. This is not a result but a method. See the previous comments.

 Line 159-160. Need editing.

*Suggestion: A total of 137, including 40 INH susceptible randomly selected from DRS, were used in detecting INH resistance in MDR (n=35) and Hr-TB (n=62) isolates.*

 Line 162. If we combine, it would be 97. Please correct it.

 Line 166. Table 1. Seems an error and the numbers are the same as table 2, check it out carefully and correct your numbers/percentages.

 Line 186. 94.3% versus 77.4%, first test it for statistical significance since you make inference in your abstract and discussion. If significant, your discussion is aligned with the location of the genes for the difference stands. If not statistically significant, you may want to update the discussion accordingly.

 Line 209. Table 5, inclusion of MDR-TB seems an error. Please correct. Again, since some are missing from the 97 (now it is 81). It needs clarification to avoid confusion.

**Discussion**

The discussion seems well written. Good job!

Line 274.  Correct ‘0.05’ as ‘0.5’.

**Conclusion**

Line 293. ‘lower…..compared’ – at the moment no strong support for this statement. If only you present it in the result with a significance test, then you may use it.

Line 295-296. Why should this be the case since the gene detected in MDR is expected to exist in Hr-TB too.  Since the difference between the two is the additional Rif-resistance. The recommendation should rather be ‘further evaluation to explain why INH resistance is less detected when INH resistance TB is detected than in MDR. You may correct it or clarify to make the readers understand it better.

**Miner comments**

Use of abbreviations – always first-time use is abbreviated; in a lot of places this is not done as it should and correct all those accordingly.

 Line 22. M should be written in full, again for first-time use.

Line 35. ‘e’ is missing in the second tuberculosis.

Line 108. Include the abbreviations, STR, ETB and PZA, since you are using them as such in the latter part of this manuscript.

Line 239. ..correct it as ‘this study suggests’ not ‘this study would suggest’

Line 256. Not encouraged to use the same word in one sentence. Replace the first ‘indicates’ with ‘shows’.

Line 261. ..Similarly, other studies…

Line 291. …any other limitations not included, see the previous comments.
