# Supplementary material for: Diagnostic performance of the GenoType MTBDRplus VER 2.0 line probe assay for the detection of isoniazid resistant Mycobacterium tuberculosis in Ethiopia
Source: PLoS One. 2023 Apr 26;18(4):e0284737. doi: 10.1371/journal.pone.0284737 (PMC10132600; doi:10.1371/journal.pone.0284737)
Supplement: Attachment — Submitted filename: Response to Reviewers.docx [file pone.0284737.s003.docx]

**Ref: PONE-D-22-26863**

**Diagnostic performance of the GenoType MTBDRplus VER 2.0 line probe assay for the detection of isoniazid resistant *Mycobacterium tuberculosis* in Ethiopia.**

**Dear Editors,**

Thank you very much for your informative comments on our manuscript entitled “Diagnostic performance of the GenoType MTBDRplus VER 2.0 line probe assay for the detection of isoniazid resistant *Mycobacterium tuberculosis* in Ethiopia”. We have addressed your comments one by one. We also appreciate you for allowing us to revise our manuscript and correct errors in the previous version. We thank the reviewers for their informative comments, and accordingly our point-by-point responses to the reviewers’ comments are given below. Also, we would like to inform you that we have used highlights to indicate where we made changes in response to the reviewers’ comments.

**Reviewers’ comments to the Author**

**Summary**

*This manuscript assesses the Diagnostic performance of the GenoType MTBDRplus VER 2.0-line probe assay for the detection of isoniazid resistant Mycobacterium tuberculosis in Ethiopia.*

*The subject remains a point of interest, however looking into the current form the manuscript seems to have major needed correction, mostly clarifying and updating the mix-ups between the methods and result section. The discussion part is well written, and a few minor edits are required there too.*

**Reviewers’ Major comments**

1. *Line 3 and 10.Order of the co-authors, we cannot have ‘e’ before ‘b’. Please replace ‘e’ with ‘b’ and correct the order accordingly.*

**Response:** Thank you for your corrective comment. It has been addressed in the revised manuscript

**Abstract:**

2. *Line 27.Introduce version 1.0 here and clarify why for version 2 LPA. Suggested reference:* [*R. N. Yadav*](https://pubmed.ncbi.nlm.nih.gov/?term=Yadav%20RN%5BAuthor%5D) *et al* [*Tuberc Respir Dis (Seoul).*](https://www.ncbi.nlm.nih.gov/pmc/articles/PMC8273024/) *2021 Jul. You may also include this part in the ‘introduction’ rather than in the abstract.*

**Response:** Thank you for your comment. It has been included in the introduction section as per your suggestion

3. *Line 29. 137 is a result, should not come here in the methods. In the methods readers are interested ‘the how part of the procedure and selections of participants.*

**Response:** Thank you for your guiding comment. The quantitative description of total isolates included in the study, which is 137, has been removed from the methods

4. *Line 35. To include your 137 here, the suggestion might look like – ‘A total of 137 isolates were identified, of those 62 were Hr-TB, 35 multidrug resistant tuberculosis (MDR-TB) and 40 INH susceptible. Remove ‘were evaluated’ this is more to go in the methods part.*

**Response:** Thank you for your suggestion. The sentence has been restated by including total number of isolates (n=137) in the result.

5. *Line 43. Replace ‘improved’ with higher since there was no intervention to claim this. Even ‘higher’ may only be used if supported by a significance test. Inference like high or low may not be used without statistical significance backing it up. P value or confidence interval, as appropriate.*

**Response:** Thank you for your critical observation. We included Fisher’s exact test to examine difference in the sensitivity between two groups and found that the sensitivity of LPA in detecting INH resistance among MDR-TB cases is significantly higher than the sensitivity of LPA in detecting INH resistance detected among Hr-TB cases (P=0.04).

6. *Line 48. In your keywords. You may remove ‘isoniazid resistant M tuberculosis’. It does not add value while it is redundant. Adding ‘Ethiopia’ does not seem to add that much too. Unless you insist better without it.*

**Response:** Thank you for your comments. We have removed ‘isoniazid resistant M tuberculosis’ and ‘Ethiopia’ from the lists of keywords

**Introduction**

7. *See the comment in the abstract on this: Introduce version 1.0 and clarify why for version 2 LPA. Suggested reference:* [*R. N. Yadav*](https://pubmed.ncbi.nlm.nih.gov/?term=Yadav%20RN%5BAuthor%5D) *et al* [*Tuberc Respir Dis (Seoul).*](https://www.ncbi.nlm.nih.gov/pmc/articles/PMC8273024/) *2021 Jul. You may also include this part in the ‘introduction’ rather than in the abstract.*

**Response:** We have compared version-1 LPA with version-2 LPA and included the merit of using version-2 over version-1. However, we used evaluation studies previously published (by Crude and Seifert) prior to the suggested article to address this comment

8. *Line 81-84. Please align this description of aim with that of the abstract and make it clearer as it seems not immediately clear on its consistency, especially with the abstract.*

**Response**: Thank you for the pertinent comment. To address this comment we have described the objective of study coherently within the paragraph and in consistence with the abstract

**Methods**

9. *Line 86. On the 137, see the same comment under abstract.*

**Response**: We have addressed this comment in similar with our response to comment #3 above.

10. *Line 90-103. This part is not really a method to address your objective, probably for the survey. Thus, it should be removed. Here, you rather describe in a succinct way the exact methods/procedures you used to help you reach to 137 (without mentioning numbers, unless required). For instance, the first paragraph of your result is mostly the methods part that should come in this section.*

**Response**: Thank you very much for your informative comments. We have addressed your comments across the methods and results section. We have described the methods in succinct way without mentioning less important numerical data of previous survey

11. *Line 136-140. Sensitivity against what? Phenotypic DST? Why do you want to measure it against weak methods, at least it is not a gold standard. If there was no alternative way to measure the test (Genotype) performance, then it should be stated as a limitation in the discussion part. I haven’t seen this in the discussion.*

**Response:** Thank you very much for pointing this out. We used phenotypic DST as a reference method. Culture-based phenotypic DST methods are currently the gold standard for drug resistance detection in *M tuberculosis*. DST using the BACTEC MGIT system is the preferred method for performing DST for many anti-TB agents particularly for detecting resistance against INH. We believe that MGIT method is very important reference method for evaluation study. We did not perform sequencing due to lack of the infrastructure for library preparation and bioinformatics. Given worthiness of using sequencing as an additional reference method, we have mentioned in the limitation part of the revised manuscript (Line 279-289).

**Results**

12. *Line 150. Include 1594 with the denominator in your abstract too, e.g., 62% (1594/2268).*

*Suggestion: ‘Phenotypic DST results of MTBC isolates were available for 62% (1594/2268) of those 3.9% (62/1594) and 2.2% (35/1594) were from Hr-TB and MDR-TB, respectively.’*

**Response:** Thank you very much for your valuable suggestion. Since the final DRS manuscript is on the write up process, as a writing team, we have observed that the total number of complete DST data have been reduced from 1594 to **1552** after data cleaning and accordingly we considered this changes as a denominator in the main manuscript and flowchart. However the total number of study participants (n=2268) remain the same

13. *Line 153-156. This is not a result but a method. See the previous comments.*

**Response**: We have addressed this comment in similar to our response to comment #3 and # 9 above.

14. *Line 159-160. Need editing.*

*Suggestion: A total of 137, including 40 INH susceptible randomly selected from DRS, were used in detecting INH resistance in MDR (n=35) and Hr-TB (n=62) isolates.*

**Response:** We adapted the above suggestion and have revised the results section

15. *Line 162. If we combine, it would be 97. Please correct it.*

Response: Thank you for your comment. Since sensitivity is expressed in proportion, we presented our result in percentage 83.5% (81/97). 97 isolates were detected as INH resistant using phenotypic DST, of which 81 isolates were detected as INH resistant using LPA and the remaining 16 INH resistant phenotypes were missed by LPA.

16. *Line 166. Table 1. Seems an error and the numbers are the same as table 2, check it out carefully and correct your numbers/percentages.*

**Response:** Thank you for your critical observation. We have revised and corrected numeric errors in Table 2 accordingly.

17. *Line 186. 94.3% versus 77.4%, first test it for statistical significance since you make inference in your abstract and discussion. If significant, your discussion is aligned with the location of the genes for the difference stands. If not statistically significant, you may want to update the discussion accordingly*.

**Response:** Thank you for your critical observation. We included Fisher’s exact test to examine difference in the sensitivity between two groups and found that the sensitivity of LPA in detecting INH resistance among MDR-TB cases is significantly higher than the sensitivity of LPA in detecting INH resistance detected among Hr-TB cases (P=0.04).

18. *Line 209. Table 5, inclusion of MDR-TB seems an error. Please correct. Again, since some are missing from the 97 (now it is 81). It needs clarification to avoid confusion.*

**Response:** Thank you for raising this point. MDR-TB was included in this table as a comparator to show the difference in the frequency and variants of INH resistance conferring mutation between MDR-TB and Hr-TB cases. As indicated in the table *kat*G315 mutation was detected in all MDR isolates compared to Hr-TB. We have re-stated in the result section that 97 isolates were detected as INH resistant using phenotypic DST of which 81 isolates were detected as INH resistant using LPA

**Discussion**

*The discussion seems well written. Good job!*

19. *Line 274. Correct ‘0.05’ as ‘0.5’.*

**Response:** Thank you for pointing this out. We have corrected it accordingly

**Conclusion**

20. *Line 293. ‘lower…..compared’ – at the moment no strong support for this statement. If only you present it in the result with a significance test, then you may use it.*

**Response:** Thank you for your pertinent comment. We included Fisher’s exact test to examine difference in the sensitivity between two groups and found that the sensitivity of LPA in detecting INH resistance among MDR-TB cases is significantly higher than the sensitivity of LPA in detecting INH resistance detected among Hr-TB cases (P=0.04)

21. *Line 295-296. Why should this be the case since the gene detected in MDR is expected to exist in Hr-TB too. Since the difference between the two is the additional Rif-resistance. The recommendation should rather be ‘further evaluation to explain why INH resistance is less detected when INH resistance TB is detected than in MDR. You may correct it or clarify to make the readers understand it better.*

**Response:** Thank you for your comment. As it has been observed in our study and others, the most common isoniazid resistant conferring mutations, Kat G and InhA tend to occur among MDR isolates more frequently than among Hr-TB isolates. It evident that INH resistance conferring genes detected in MDR is expected to exist in Hr-TB too, because it is Hr-TB that develops into MDR-TB. However all Hr-TB does not develop into MDR-TB, this is particularly apparent with Hr-TB cases that develop INH resitance mutation other than KatG and InhA.

**Minor comments**

22. *Use of abbreviations – always first-time use is abbreviated; in a lot of places this is not done as it should and correct all those accordingly.*

*Line 22. M should be written in full, again for first-time use.*

*Line 35. ‘e’ is missing in the second tuberculosis.*

*Line 108. Include the abbreviations, STR, ETB and PZA, since you are using them as such in the latter part of this manuscript.*

*Line 239. ..correct it as ‘this study suggests’ not ‘this study would suggest’*

*Line 256. Not encouraged to use the same word in one sentence. Replace the first ‘indicates’ with ‘shows’.*

*Line 261. ..Similarly, other studies…*

*Line 291. …any other limitations not included, see the previous comments.*

**Response:** Thank you for the pertinent comment and suggestion. We have addressed all minor comments as per your suggestion across the manuscript.

Finally, we would like to thank the reviewers once more for sparing the time to write so many detailed and useful comments.
