# Supplementary material for: Diagnostic performance of the GenoType MTBDRplus VER 2.0 line probe assay for the detection of isoniazid resistant Mycobacterium tuberculosis in Ethiopia
Source: PLoS One. 2023 Apr 26;18(4):e0284737. doi: 10.1371/journal.pone.0284737 (PMC10132600; doi:10.1371/journal.pone.0284737)
Supplement: Attachment — Submitted filename: Response to Reviewers.docx [file pone.0284737.s005.docx]

**Ref: PONE-D-22-26863**

**Diagnostic performance of the GenoType MTBDRplus VER 2.0 line probe assay for the detection of isoniazid resistant *Mycobacterium tuberculosis* in Ethiopia.**

**Dear Editors,**

Thank you very much for your informative comments on our manuscript entitled “Diagnostic performance of the GenoType MTBDRplus VER 2.0 line probe assay for the detection of isoniazid resistant *Mycobacterium tuberculosis* in Ethiopia”. We have addressed your comments one by one. We also appreciate you for allowing us to revise our manuscript and correct errors in the previous version. We thank the reviewers for their informative comments, and accordingly our point-by-point responses to the reviewers’ comments are given below.

**Reviewer #2:** Thank you for recommending our manuscript for publication.

**Reviewer #4:**

**Major comments to the Author**

1. This study did not provide any information about the respective prevalence of isoniazid resistance among rifampicin-susceptible isolates and rifampicin-resistant isolates.

***Response:*** *Thank you for your comments. The global prevalence of Hr-TB (M tuberculosis isolates resistant to isoniazid but susceptible to rifampicin) was presented in the background section (line # 56). According to the DRS, which is source of our study, Hr-TB prevalence in our setting (Ethiopia) was 4.0% (62/1552) presented in result section line # 152. Regarding prevalence of isoniazid resistance among rifampicin-resistant isolates, we detected INH resistance in 35 of 37 RIF resistant isolates (94.6%) and presented in line # 153. We have used this prevalence (94.6%) as the pre-test probability of isoniazid resistance among rifampicin-resistant isolates to calculate the PPV and NPV of the LPA in our study.*

2. It is uncertain whether the 40 randomly selected isoniazid-susceptible isolates were rifampicin-susceptible. It may be better to evaluate the specificity of Hain ver. 2 for isoniazid resistance using isoniazid-susceptible isolates with matched bacillary susceptibility to rifampicin among rifampicin-susceptible and rifampicin-resistant isolates, respectively.

***Response:*** *Thank you for your comments. It has been presented in the Fig-1 that the 40 randomly selected isoniazid-susceptible isolates are rifampicin-susceptible (see Figure 1). Rif mono-resistant were excluded. We did matching based on phenotypic susceptibility to both isoniazid and rifampicin. Matching susceptibility for other anti-TB drugs could be difficult as resistance develop sequentially such that most of Hr-TB tend to develop to MDR-TB while MDR tend to develop to XDR. Moreover matching for other drugs have little implication to recommend/refute LPA the program and clinical management as classification of Hr-TB and MDR-TB is based solely on bacillary susceptibility to isoniazid and/or rifampicin.*

3. PPV and NPV were erroneously estimated in this study without due consideration of the pre-test probability of isoniazid resistance. The study sample of 62 isoniazid-resistant and rifampicin-susceptible isolates plus an arbitrarily determined number of randomly selected isoniazid-susceptible isolates would not give any information about the pre-test probability of isoniazid resistance among rifampicin-susceptible isolates. Neither would the study sample of 35 MDR isolates plus the same batch of isoniazid-susceptible isolates give any information about the pre-test probability of isoniazid resistance among rifampicin-resistant isolates.

***Response:*** *Thank you for your valuable comments. The pre-test probability of isoniazid resistance has been determined by using DRS data. Cases whose phenotypic DST is available was 1552. Accordingly*

- *The pre-test probability of isoniazid among rifampicin-susceptible isolates was 4.1% (62/1515). We revised NPV= 99.0% (98.5-99.4)*
- *Pre-test probability of isoniazid resistance among rifampicin-resistant isolates was 94.6% (35/37) = We revised NPV=50.0% (20.6-79.3)*
- *Pre-test probability of isoniazid resistance among total isolates (in survey) was 6.25% (97/1552). We revised NPV = 98.9% (98.3% to 99.3%)*

4. The performance of Hain ver.2 is heavily dependent on the pre-test probability of isoniazid resistance. This is why WHO has recommended that phenotypic DST should be done when Hain ver. 2 does not detect isoniazid resistance and the pre-test probability of isoniazid resistance is high. In general, isoniazid resistance is highly correlated with rifampicin resistance. It may be reasonable to expect a prevalence of isoniazid resistance in the range of 5-15% among rifampicin-susceptible TB cases, and about 90% among rifampicin-resistant TB cases. In a theoretical population of 1000 rifampicin-susceptible TB patients, with a pre-test probability of 15% for isoniazid resistance, if Hain ver. 2 is 77.4% sensitive and 100% specific, Hain ver. 2 would detect 116 cases and miss 34 cases. There are no false-positive cases and 850 true-negative cases. PPV is 100% and NPV is 96.2%. On the other hand, in a theoretical population of 1000 rifampicin-resistant TB patients, with a pre-test probability of 90% for isoniazid resistance, if Hain ver. 2 is 94.3% sensitive and 100% specific, Hain ver. 2 would detect 849 cases and miss 51 cases. There are no false-positive cases and 100 true-negative cases. PPV is 100% and NPV is 66.2%. Thus, with reference to sensitivity and specificity values found in this study, Hain ver. 2 has excellent PPV for isoniazid-resistant case regardless of rifampicin susceptibility, and a more reliable negative result when the patient has rifampicin-susceptible TB than when the patient has MDR-TB.

***Response:*** *Thank you for your valuable comments. We completely accept the explanation provided in terms of PPV and NPV. However, with disease like Hr-TB that has high negative impact on population and patient, we believe that the sensitivity and the specificity of index test (i.e LPA) should be the primary parameter for evaluating a diagnostic tool prior to assessing the PPV or PPV for its implementation in specific settings. Missing a single case of Hr-TB could fuel its transmission, amplification to MDR-TB and may result in poor treatment outcome. In our study we found sensitivity of 77.4%, which implies that about a quarter (~22.6%) of Hr-TB will be missed with LPA ver.2. Currently available evidence suggest that approximately 94% of strains that are missed by MTBDRplus v2 LPA could be detected by covering more DNA adjacent to existing target regions (See* [*https://www.ncbi.nlm.nih.gov/pmc/articles/PMC6153830/*](https://www.ncbi.nlm.nih.gov/pmc/articles/PMC6153830/)*). In the same line, due to the low sensitivity of old MTBDR LPA assay in detecting INH resistance among MDR, the manufacturer improved its sensitivity for detecting INH* ***among MDR-TB*** *by developing GenoType MTBDR****plus*** *assay by incorporating additional gene (i.e. the regulatory region of inhA) (Reference doi:10.1128/JCM.00521-07). Given high chances of amplification to MDR-TB, treatment failure of Hr-TB if undiagnosed, we believe that having a diagnostic tool that detect (rule in) majority of Hr-TB (>90%) is much more prioritized than a tool which have only an excellent PPV and specificity.*

5. It has been shown that, if the TB treatment regimen is properly formulated with inclusion of ethambutol, missing initial isoniazid resistance does not affect the treatment success rate among rifampicin-susceptible TB cases treated with rifampicin, ethambutol and pyrazinamide for six months in comparison with drug susceptible TB treated with the standard six-month regimen. The situation is very different when rifampicin resistance is missed.

***Response:*** *Thank you for your comment. We agree that early detection of RIF is very important. However, there is a growing body of literature that recognizes initial isoniazid resistance is associated with poorer treatment outcome compared to drug susceptible TB (DS-TB). Accordingly, WHO conditionally recommended levofloxacin, instead of isoniazid, to treat Hr-TB cases in combination with rifampicin, ethambutol and pyrazinamide (*[*https://www.who.int/publications/i/item/9789241550079*](https://www.who.int/publications/i/item/9789241550079)*). Due to very low certainty in the estimates of effects, we believe that feature research is needed to revise or continue with the treatment recommendation. Despite this, early of detection of Hr-TB is very important to prevent further development to MDR-TB then XDR-TB. We have explained in, the background section of the manuscript, the clinical and programmatic implication of missing cases with initial isoniazid resistance.*

6. There may be a typo error regarding the *P value* (line 39 in abstract, line 172 in results). The *P value* was probably 0.04 rather than 0.4.

***Response:*** *Thank you for your corrective comment. It has been addressed in the revised manuscript (line 39 in abstract, line 172 in results).*

7. In summary, study findings did not appear to substantiate the conclusion.

***Response:*** *Thank you for your comment. Major findings that support each conclusion has been presented point-by-point below.*

Conclusion 1: GenoType MTBDRplus VER 2.0 LPA demonstrated improved performance in detecting INH resistance among MDR-TB cases compared to Hr-TB cases.

*Findings (line 169-172)*: The assay detected only 48 of the 62 Hr-TB identified by phenotypic DST resulting in a sensitivity of 77.4% (95% CI: 65.0- 87.1). The sensitivity of LPA in detecting INH resistance among Hr-TB cases was significantly less than that of detecting INH resistance among MDR-TB cases (77.4% vs 94.3%, *P*= 0.04).

Conclusion 2: The *kat*G315 mutation is the most common INH resistance conferring gene among Hr-TB and MDR-TB isolates.

*Findings (line 201, 211 and 212): kat*G315 mutation accounted for 91.7% (n=44) (among Hr-T B isolates).

Finding *(Line 211 and 212): KatG315* mutations accounted for all (100%) of mutations detected among MDR isolates.

Conclusion 3 (that is a recommendation): Additional INH resistance conferring mutations should be evaluated to improve the sensitivity of the GenoType MTBDRplus VER 2.0 for the detection of INH resistance among Hr-TB cases.

*Findings (line 169-172)*: The assay detected only 48 of the 62 Hr-TB identified by phenotypic DST resulting in a sensitivity of 77.4% (95% CI: 65.0- 87.1). The sensitivity of LPA in detecting INH resistance among Hr-TB cases was significantly less than that of detecting INH resistance among MDR-TB cases (77.4% vs 94.3%, *P*= 0.04). Since assay missed 22.6% of Hr-TB cases, which is significantly high, we recommend evaluation of additional resistance genes to increase the sensitivity of the assay. Currently available evidence suggest approximately 94% of strains that are missed by MTBDRplus v2 LPA could be detected by covering more DNA adjacent to existing target regions (See https://www.ncbi.nlm.nih.gov/pmc/articles/PMC6153830/). Therefore, we recommend the manufacturer to evaluate and incorporate additional resistance genes to increase the sensitivity of the assay.

**Minor comments**

1. The Clopper-Pearson exact method was probably used in estimating the 95% confidence interval. The modified Wald method might be more accurate.

***Response****: Thank you for recommending more accurate method. It has been addressed in the revised manuscript*

**Reviewer #5:**

**Minor stylistic comments for consideration**.

Line 69: Please add “the” before “added.”

Lines 78-79: Suggest “…, can additionally be performed on smear-negative clinical specimens given its higher sensitivity than version 1.0.”

Line 116: Replace “was” with “were.”

***Response:*** *Thank you for the editorial comment and for recommending our manuscript for publication. We have addressed all comments as per your suggestion across the manuscript (i.e. line 69, 78-79, and 116)*
